# Investigation of poly(γ-glutamic acid) production via online determination of viscosity and oxygen transfer rate in shake flasks

**DOI:** 10.1186/s13036-017-0065-4

**Published:** 2017-07-12

**Authors:** Lena Regestein née Meissner, Julia Arndt, Thomas G. Palmen, Tim Jestel, Hitoshi Mitsunaga, Eiichiro Fukusaki, Jochen Büchs

**Affiliations:** 1 AVT - Biochemical Engineering, RWTH Aachen University, Forckenbeckstr. 51, 52074 Aachen, Germany; 2 AVT - Enzyme Process Technology, RWTH Aachen University, Forckenbeckstr. 51, 52074 Aachen, Germany; 3 Department of Biotechnology, Graduate School of Engineering, Osaka University, 2-1 Yamadaoka, Suita, Osaka, 565-0871 Japan

**Keywords:** *Bacillus licheniformis*, Online volumetric power input, Effective viscosity, Shake flask

## Abstract

**Background:**

Poly(γ-glutamic acid) (γ-PGA) is a biopolymer with many useful properties making it applicable for instance in food and skin care industries, in wastewater treatment, in biodegradable plastics or in the pharmaceutical industry. γ-PGA is usually produced microbially by different *Bacillus* spp. The produced γ-PGA increases the viscosity of the fermentation broth. In case of shake flask fermentations, this results in an increase of the volumetric power input. The power input in shake flasks can be determined by measuring the torque of an orbitally rotating lab shaker. The online measurement of the volumetric power input enables to continuously monitor the formation or degradation of viscous products like γ-PGA. Combined with the online measurement of the oxygen transfer rate (OTR), the respiration activity of the organisms can be observed at the same time.

**Results:**

Two different *Bacillus licheniformis* strains and three medium compositions were investigated using online volumetric power input and OTR measurements as well as thorough offline analysis. The online volumetric power input measurement clearly depicted changes in γ-PGA formation due to different medium compositions as well as differences in the production behavior of the two investigated strains. A higher citric acid concentration and the addition of trace elements to the standard medium showed a positive influence on γ-PGA production. The online power input signal was used to derive an online viscosity signal which was validated with offline determined viscosity values. The online measurement of the OTR proved to be a valuable tool to follow the respiration activity of the cultivated strains and to determine its reproducibility under different cultivation conditions.

**Conclusions:**

The combination of the volumetric power input and the OTR allows for an easy and reliable investigation of new strains, cultivation conditions and medium compositions for their potential in γ-PGA production. The power input signal and the derived online viscosity directly reflect changes in γ-PGA molecular weight and concentration, respectively, due to different cultivation conditions or production strains.

**Electronic supplementary material:**

The online version of this article (doi:10.1186/s13036-017-0065-4) contains supplementary material, which is available to authorized users.

## Background

Poly(γ-glutamic acid) (γ-PGA) is an anionic, water-soluble, biodegradable, edible, and nontoxic biopolymer. It consists of up to 10,000 D- and/or L-glutamic acid monomers and can be characterized by its molecular weight distribution and the ratio of D- to L-glutamic acid subunits. In contrast to proteins where amino acids are bound by α-amide linkages, glutamic acid monomers in γ-PGA are linked by γ-peptide bonds. This property makes γ-PGA stable against proteases. γ-PGA is mainly produced by *Bacillus* spp. and can either be degraded chemically or enzymatically by specific depolymerases. These depolymerases are often expressed by the producing organisms themselves. Microbially produced γ-PGA by *Bacillus* spp. has generally a high molecular weight in the range of 10-1000 kDa and a broad polydispersity [1–3]. Originally known from the Japanese food natto, today a great variety of possible applications for γ-PGA exists: in food and skin care industries [4–6], in wastewater treatment [7, 8], in biodegradable plastics [9], as cryo-protectant [10], and in the pharmaceutical industry as drug delivery systems, hydrogels, and surgical glue [2, 11–13].

The specific power input per liquid volume is one of the essential parameters for the scale-up and scale-down of microbial processes. Parameters which directly have an impact on biological cultures such as oxygen supply, carbon dioxide removal, degree of mixing as well as hydromechanical stress or dispersion of an organic liquid phase are all more or less dependent on the specific power input [14]. Besides shaking frequency, flask size, and filling volume, the viscosity of the fermentation broth is one of the factors influencing the power input in unbaffled shake flasks [15, 16]. As shaking frequency, flask size, and filling volume are usually constant throughout an experiment, a temporal alteration in viscosity is the only variable factor influencing the power input during fermentation in shake flasks. With increasing viscosity, the power input also increases and vice versa. The power input in shake flasks can be determined by measuring the torque of an orbital rotating lab shaker with a torque sensor integrated in the drive of the shaker, a method first introduced by Büchs et al. [15]. Temporal alterations in viscosity during fermentation processes can be due to filamentous biomass formation or biopolymer production and degradation. The method of online power input measurement to follow biomass or product formation was already successfully applied for different fermentation systems, including the filamentous fungus *Trichoderma reseei,* the xanthan producer *Xanthomonas campestris* [17], and *Azobacter vinelandii* producing alginate [18]. The method was also used for the optimization of culture conditions in shake flasks to especially prevent unfavorable “out-of-phase” conditions which strongly impair oxygen supply and mixing [14, 19, 20].

No online measurement signal of power input and viscosity as indicators for γ-PGA production in shake flasks are published so far. Online signals of the oxygen transfer rate (OTR) and the volumetric power input enable to continuously monitor respiration activity and product formation and easily investigate new strains, cultivation conditions and medium compositions for their potential in γ-PGA production.

## Methods

**Fig. 1:**
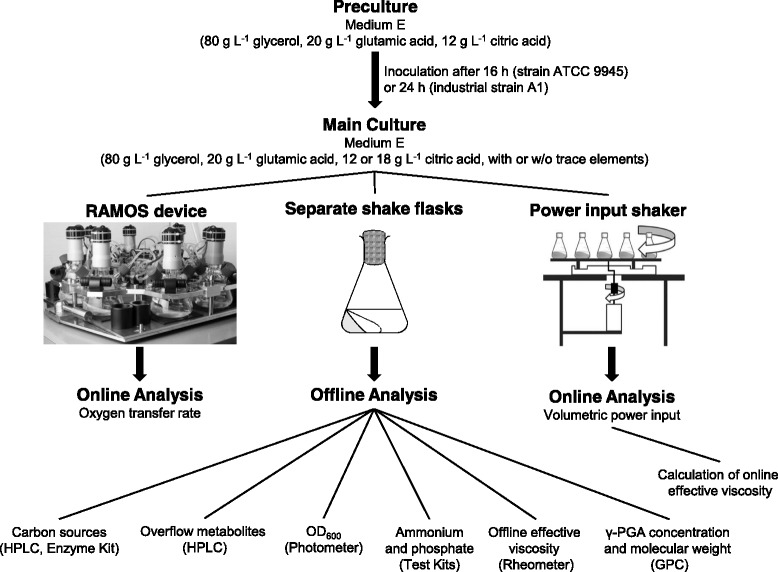
Overview of different cultivation steps, cultivation systems, and measured online and offline data. Both, pre-culture and main culture, were performed in mineral medium E containing glycerol, glutamic acid, and citric acid as carbon sources. Pre-cultures were carried out in 500 mL shake flasks in a RAMOS (Respiration Activity MOnitoring System) device. The main culture was performed in 500 mL flasks in three cultivation systems in parallel: RAMOS device, power input shaker and separate 500 mL shake flasks. The RAMOS device allowed for an online measurement of the oxygen transfer rate (OTR) of the culture. The power input shaker contained a torque sensor for online determination of the volumetric power input which was used to calculate the online effective viscosity. Separate shake flasks were used for offline analysis

An overview of the cultivation procedure, the different applied cultivation systems and the determined online and offline data for all experiments presented in this study is given in Fig. 1. A detailed description of the applied techniques can be found in the following paragraphs.

### Microbial strains and medium

The strain *Bacillus licheniformis* ATCC 9945 was acquired from the *American Type Culture Collection* and stored as glycerol stocks in medium E [21] with 30% (*w*/*v*) glycerol at −80 °C. The industrial strain *Bacillus licheniformis* A1 was originally used as a protease producer which secreted γ-PGA as an undesired by-product [22, 23]. It was kindly provided by Henkel AG & Co. KGaA (Düsseldorf, Germany). The strain was stored as glycerol stocks in complex LB medium (5 g L^−1^ yeast extract, 10 g L^−1^ tryptone, 10 g L^−1^ NaCl, pH 7.0) with 30% (*w*/*v*) glycerol at −80 °C. Strain A1 is resistant to kanamycin. Therefore, kanamycin sulfate was added to the media with a final concentration of 50 mg L^−1^.

All chemicals were purchased from Carl Roth GmbH & Co. KG (Karlsruhe, Germany), Merck KGaA (Darmstadt, Germany), VWR International GmbH (Darmstadt, Germany), or AppliChem GmbH (Darmstadt, Germany), and were of analytical grade.

For pre-culture and main culture, the same medium was used, namely the mineral medium E [21] which contained per liter: 80 g glycerol, 20 g L-glutamic acid, 12 g citric acid, 7 g (NH_4_)_2_SO_4_, 0.5 g K_2_HPO_4_, 0.5 g MgSO_4_⋅7H_2_O, 0.04 g FeCl_3_⋅6H_2_O, 0.15 g CaCl_2_⋅2H_2_O, 0.104 g MnSO_4_⋅H_2_O. For some experiments, the concentration of citric acid was increased from 12 to 18 g L^−1^ or a trace elements stock solution was added to the medium after autoclaving (preparation see below). The pH value of the medium was set to 7.4 with 5 M NaOH before autoclaving. The medium was sterilized for 20 min at 121 °C. Prior to inoculation, the pH value was checked and, if necessary, re-adjusted to pH 7.4 with 5 M HCl or NaOH. A 500× trace elements stock solution was prepared and applied for some experiments, containing per liter: 530 mg CoCl_2_⋅6H_2_O, 260 mg ZnCl_2_, 10 mg H_3_BO_3_, 660 mg NiSO_4_⋅6H_2_O, 310 mg CuSO_4_⋅5H_2_O, and 650 mg Na_2_MoO_4_⋅2H_2_O. The trace elements stock solution was diluted 1:5 with distilled water to obtain a 100× stock solution, sterile filtered and used for medium preparation (10 mL trace elements stock solution per liter medium).

### Cultivation conditions and online measurement of oxygen transfer rates (OTR)

A one-stage pre-culture was used to inoculate main cultures. The pre-culture was inoculated with 0.4% (*v*/*v*) from glycerol stock and cultivated for 16 h (strain ATCC 9945) or 24 h (strain A1). For main cultures, a master mix was prepared by inoculating the medium with the pre-culture to obtain an initial OD_0_ = 0.05 at 600 nm and then the desired filling volume was transferred to modified Erlenmeyer (RAMOS) flasks and to normal Erlenmeyer flasks with cotton plugs for sampling and online power input measurement. Pre-culture and main culture were both cultivated under the following conditions: 500 mL shake flasks, filling volume 100 mL, shaking frequency 250 rpm, shaking diameter 50 mm, temperature 37 °C. Online monitoring of the oxygen transfer rates (OTR) of pre-culture and main culture was realized using a Respiration Activity Monitoring System (RAMOS) [24, 25]. Commercial versions are available from Kuhner AG, Birsfelden, Switzerland or HiTech Zang, Herzogenrath, Germany.

The OTR was calculated from the measurement of the oxygen partial pressure in the head space of the RAMOS shake flasks. The equation for the OTR calculation is given by eq. 1, with the moles of oxygen *n*_*O2*_ (mol), the liquid filling volume of the RAMOS shake flask *V*_*fl*_ (L), the time *t* (h), the difference of oxygen partial pressure *Δp*_*O2*_ (bar), the time of the measuring phase *Δt* (h), the gas volume of the RAMOS shake flask *V*_*g*_ (L), the gas constant *R* = 8.314 (J mol^−1^ K^−1^), and the temperature *T* (K):

1 $$ OTR=\frac{nO2}{Vfl\cdot t}=\frac{\Delta pO2}{\Delta t}\cdot \frac{Vg}{R\cdot T\cdot Vfl} $$

The aeration rate in the RAMOS device is adjusted in such a way that the head space concentration of the gaseous compounds is equal to that of an Erlenmeyer shake flask with a cotton plug, which is aerated by diffusion. The suitability of the RAMOS technique also for viscous fermentation broths has been verified [17, 19, 26]. RAMOS flasks, sampling flasks, and flasks for the online power input measurement were filled from the same master mix and cultivated in parallel and under identical conditions to guarantee that cultures ran synchronously. Erlenmeyer flasks withdrawn for sampling were not placed back on the shaker.

### Online power input measurement

The online measurement of power input was performed with a special lab shaker with a torque sensor integrated in the drive of the shaker, as described before [15–17]. The shaker is a modified form of a shaker from Kühner AG (Type LS-X, Birsfelden, Switzerland). A motor drive (Visco-Pakt rheo-110, Hitec Zang GmbH, Herzogenrath, Germany) enables the adjustment and control of the shaking frequency as well as continuous torque readout (sampling rate: 5 s^−1^). From the data of the continuous torque readout, an average value was calculated every 12 min (5 measuring points per hour). Reference measurements were conducted in order to determine and consider the influence of friction losses due to mechanical friction in the ball bearing of the shaker and aerodynamic resistance of the shake flasks. For these reference measurements, the liquid was replaced by a solid mass with the same weight as the liquid. Therefore, the pure torque values, resulting from the friction between rotating liquid and shake flask wall, could be determined. The online volumetric power input ^*P*^ */*_*VL*_ (W m^−3^) can be calculated from the measured torque signal *M* (N m), the filling volume *V*_*L*_ (m^3^), and the shaking frequency *n* (s^−1^) with Eq. 2 [27]:

2 $$ \frac{P}{V_L}=2\cdot \uppi \cdot n\cdot \frac{M}{V_L}=2\cdot \uppi \cdot n\cdot \frac{M_{liquid}-{M}_{solid}}{V_L} $$

Additionally, the evaporation from the shake flasks during the experiments was determined gravimetrically and the reduction in filling volume was considered when calculating the volumetric power input.

### Offline viscosity measurements

The viscosity of the fermentation broth was measured offline with a Physica MCR301 rheometer (Anton Paar Germany GmbH, Ostfildern-Scharnhausen, Germany) in a range of shear rates between 10 s^−1^ and 3000 s^−1^. The rheometer was equipped with a cone-plate measuring system from Anton Paar (cone CP50-0.5/TG with cone diameter 49.945 mm, cone angle 0.467°, and cone truncation 54 μm; plate P-PTD200/TG + H-PTD200). Data analysis was performed with the software RheoPlus/32 V3.40 (Anton Paar). 480 μL of fresh, untreated sample were used for viscosity measurements at 37 °C. As γ-PGA solutions exhibit pseudo-plastic properties, the effective viscosity depends on the shear rate which is present in the shake flask at the time point of sampling. Therefore, effective shear rate and effective viscosity were calculated for each viscosity measurement of each sample according to the equation recently published by Giese et al. [17]. Viscosity measurements were chosen as an additional, simple and general method to follow γ-PGA formation and degradation during the cultivation besides gel permeation chromatography and to validate the online viscosity calculated from the online power input signal.

### Calculation of online viscosity

The online power input data were used to calculate an online viscosity signal. This calculation is conducted by calculating first the dimensionless modified Newton number *Ne′* with the power input *P* (W), the density *ρ* (kg m^−3^), the shaking frequency *n* (s^−1^), the flask diameter *d* (m), and the filling volume *V*_*L*_ (m^3^) using Eq. 3 [16]:

3 $$ N{e}^{\prime }=\frac{P}{\rho \bullet {n}^3\bullet {d}^4\bullet {V}_L^{1/3}} $$

The Newton-Reynolds correlation introduced by Büchs et al. [16] can be used to iteratively calculate the flask Reynolds number *Re* (−) from the modified Newton number *Ne*′ (−) with Eq. 4:

4 $$ N{e}^{\prime }=70\bullet {Re}^{-1}+25\bullet {Re}^{-0.6}+1.5\bullet {Re}^{-0.2} $$

From the flask Reynolds number, finally the viscosity *η* (Pa s) can be calculated with the density *ρ* (kg m^−3^), the shaking frequency *n* (s^−1^), and the flask diameter *d* (m) using Eq. 5 [16]:

5 $$ \mathit{\operatorname{Re}}=\frac{\rho \bullet n\bullet {d}^2}{\eta} $$

**Fig. 2:**
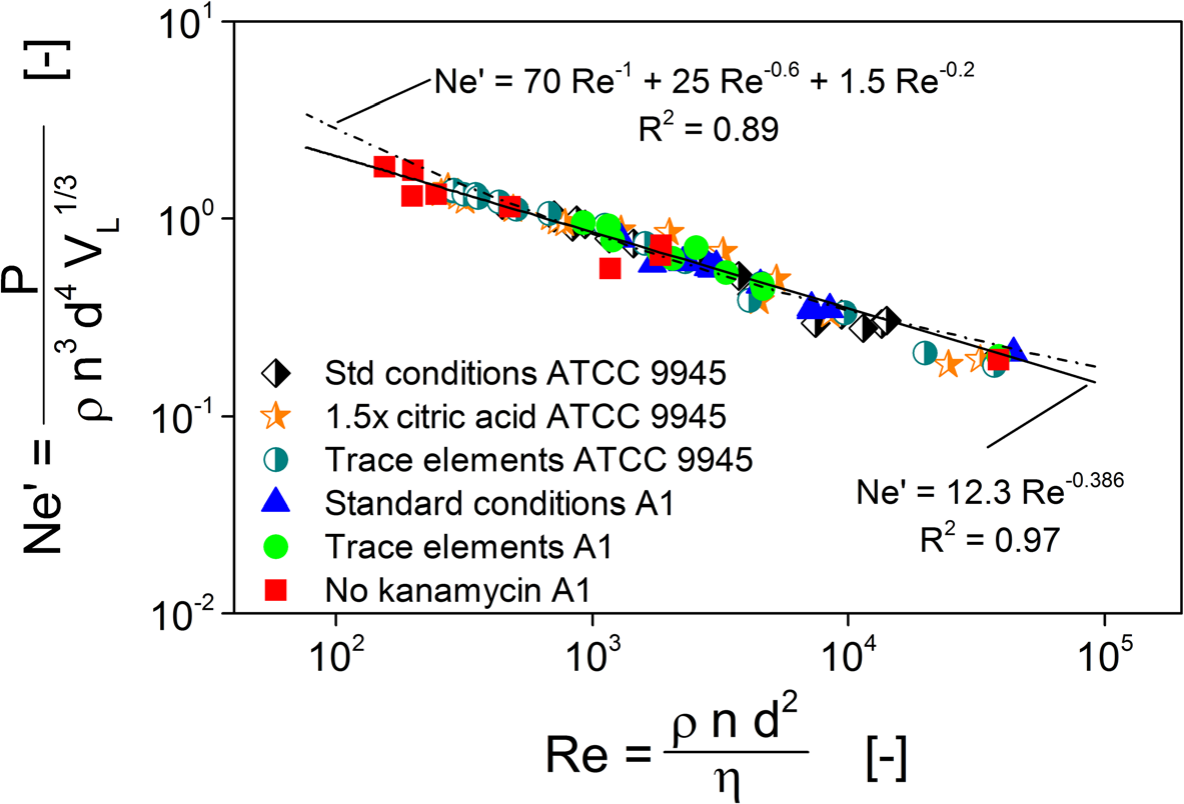
Presentation of all measuring points of the offline viscosity measurements for all experiments presented in this study in form of the modified Newton number (*Ne′*) dependent on the flask Reynolds number (*Re*). The *Re* number was calculated directly from the measured offline viscosity using Eq. 5 while the *Ne′* number was calculated with Eq. 3 from the online determined power inputs measured at the same time points at which the samples for viscosity measurements were taken. The general Newton-Reynolds correlation (Eq. 4) of Büchs et al. [16] as well as the reduced Newton-Reynolds correlation established with the data in this work (Eq. 6) are also shown

Figure 2 depicts all measuring points of the offline viscosity measurements for all experiments presented in this study in form of the modified Newton number (*Ne′*) dependent on the flask Reynolds number (*Re*). The *Re* number was calculated directly from the measured offline viscosity using Eq. 5 while the *Ne′* number was calculated with Eq. 3 from the online determined power inputs measured at the same time points at which the samples for viscosity measurements were taken. Figure 2 also shows the general Newton-Reynolds correlation (Eq. 4) of Büchs et al. [16].

Equation 4 fits the data from this study reasonably well. However, a small deviation is visible. Equation 4 slightly overestimates the power introduced. This deviation is not astonishing, as Eq. 4 was fitted into data of more than 1000 different operating conditions, varying flask size, viscosity, shaking diameter, shaking frequency, and filling volume. The three additive terms in Eq. 4 were used to take account of the laminar, intermediate, and turbulent flow regime [16]. To obtain a higher precision of the calculated online viscosity in this work, a new fit was established in the limited range of operating conditions used here, with *P* (W) and *η* (Pa s) being the only variables. Since the range of the Reynolds number is limited, only one Reynolds term was used. The reduced Newton-Reynolds correlation established with the data from this study is given by Eq. 6 and also depicted in Fig. 2:

6 $$ N{e}^{\prime }=12.3\bullet {Re}^{-0.386} $$

The calculated online viscosities shown in the results section were therefore not calculated using Eq. 4, but with Eq. 6. An exemplary curve of an online power input measurement and the resulting calculated online viscosity is shown in Additional file 1: Figure S1 (supplementary material).

### Poly(γ-glutamic acid) concentration and molecular weight

Concentration and mass average molecular weight (M_w_) of γ-PGA was determined by gel permeation chromatography (GPC). The GPC was equipped with a TSKgel GMPWxl column (300 × 7.8 mm, Tosoh Bioscience GmbH, Griesheim, Germany) and a TSKgel PWxl guard column (40 × 6 mm, Tosoh Bioscience GmbH, Griesheim, Germany). The column was eluted with 30 mM KNO_3_ at 40 °C at a flow rate of 1.0 mL min^−1^. Peaks were detected with the RI detector of the integrated EcoSEC HLC-8320GPC system (Tosoh Bioscience GmbH, Griesheim, Germany). Data analysis was performed with the software WinGPC UniChrom (PSS Polymer Standards, Service GmbH, Mainz, Germany). Poly(ethylene oxide) standards with peak molecular weights (M_p_) in the range of 56 to 1015 kDa (PSS Polymer Standards, Service GmbH, Mainz, Germany) were used to create a calibration curve for molecular weight. γ-PGA sodium salt (cosmetic grade; kindly provided by Henkel AG & Co. KGaA, Düsseldorf, Germany) was used to create a calibration curve to determine concentrations. Culture broth was diluted 1:50 with distilled water and filtered with 0.2 μm filters (Bulk GHP Acrodisc 13 mm syringe filter, GHP membrane, N° 4567, Pall Life Sciences, Dreieich, Germany). 30 μL of the standards and of the filtrated samples were injected.

### Optical density

Biomass was determined by optical density measurement (OD) at 600 nm (Genesys 20 Visible Spectrophotometer, Thermo Scientific, Waltham, U.S.A.). Samples were diluted appropriately (between 1:2 and 1:50) with 0.9% NaCl solution. 0.9% NaCl solution was also used as blank. For each sample, the optical density was determined in duplicate. Cell dry weight (CDW) was also determined but was found to be strongly influenced by γ-PGA formation. For studies, in which exact CDW data are essential, e.g. to calculate mass balances for fermentation processes, a correlation between OD and CDW could be set up by using a non-γ-PGA producing *B. licheniformis* strain. However, since exact biomass concentrations were not essential for this study, only OD was used, which does not give exact concentrations but still reflect the changes in biomass over time.

### Glycerol, citric acid and overflow metabolites

Concentrations of glycerol, citric acid and overflow metabolites (acetate, acetoin, 2,3-butanediol) were determined from the supernatant of centrifuged culture broth by HPLC. If necessary, the supernatant was diluted with distilled water (1:4 or 1:10). Samples were sterile filtered with 0.2 μm filters (Rotilabo syringe filters Mini-Tip, cellulose acetate membrane, N° PP52.1, Carl Roth GmbH & Co. KG, Karlsruhe, Germany). The HPLC (Ultimate 3000, Dionex, USA) was equipped with an Organic Acid-Resin-Column (250 × 8 mm, CS-Chromatographie Service GmbH, Langerwehe, Germany) and an Organic Acid-Resin-Precolumn (40 × 8 mm, CS-Chromatographie Service GmbH, Langerwehe, Germany). The column was eluted with 5 mM H_2_SO_4_ at 60 °C at a flow rate of 0.8 mL min^−1^. Peaks were detected with a Shodex RI-101 refractometer (Showa Denko Europe, Germany). Data analysis was performed with the software Chromeleon 6.2 (Dionex, Germany).

### L-glutamic acid

Concentration of L-glutamic acid was measured from the supernatant of centrifuged culture broth with a commercially available enzyme test kit (L-Glutamic Acid Assay Kit, Megazyme International Ireland, Bray, Co. Wicklow, Ireland). Samples were treated and diluted as indicated in the manual. The assays were performed in transparent 96-well microtiter plates (Rotilabo microtest plates, F-profile (flat bottom), N° 9293.1, Carl Roth GmbH & Co. KG, Karlsruhe, Germany) in a microtiter plate reader (Synergy 4, BioTek, Winooski, VT, U.S.A.). The assays were conducted at a temperature of 25 °C. Samples were measured in triplicate.

### Ammonium and phosphate

Concentration of ammonium and phosphate ions were determined photometrically from the supernatant of centrifuged culture broth using commercially available test kits (ammonium: 2.0-150 mg L^-1^ NH_4_-N/2.6-193 mg L^-1^ NH_4_^+^, N°1,006,830,001; phosphate: 1.0-100.0 mg L^-1^ PO_4_-P/3-307 mg L^-1^ PO_4_^3-^/2-229 mg L^-1^ P_2_O_5_, N°1,007,980,001, Spectroquant®, Merck KGaA, Darmstadt, Germany). The assays were conducted according to the manufacturer’s manual. Samples were appropriately diluted with distilled water to be in the measuring range of the assays.

## Results and discussion

### Cultivation of *Bacillus licheniformis* ATCC 9945 in standard mineral medium E

**Fig. 3:**
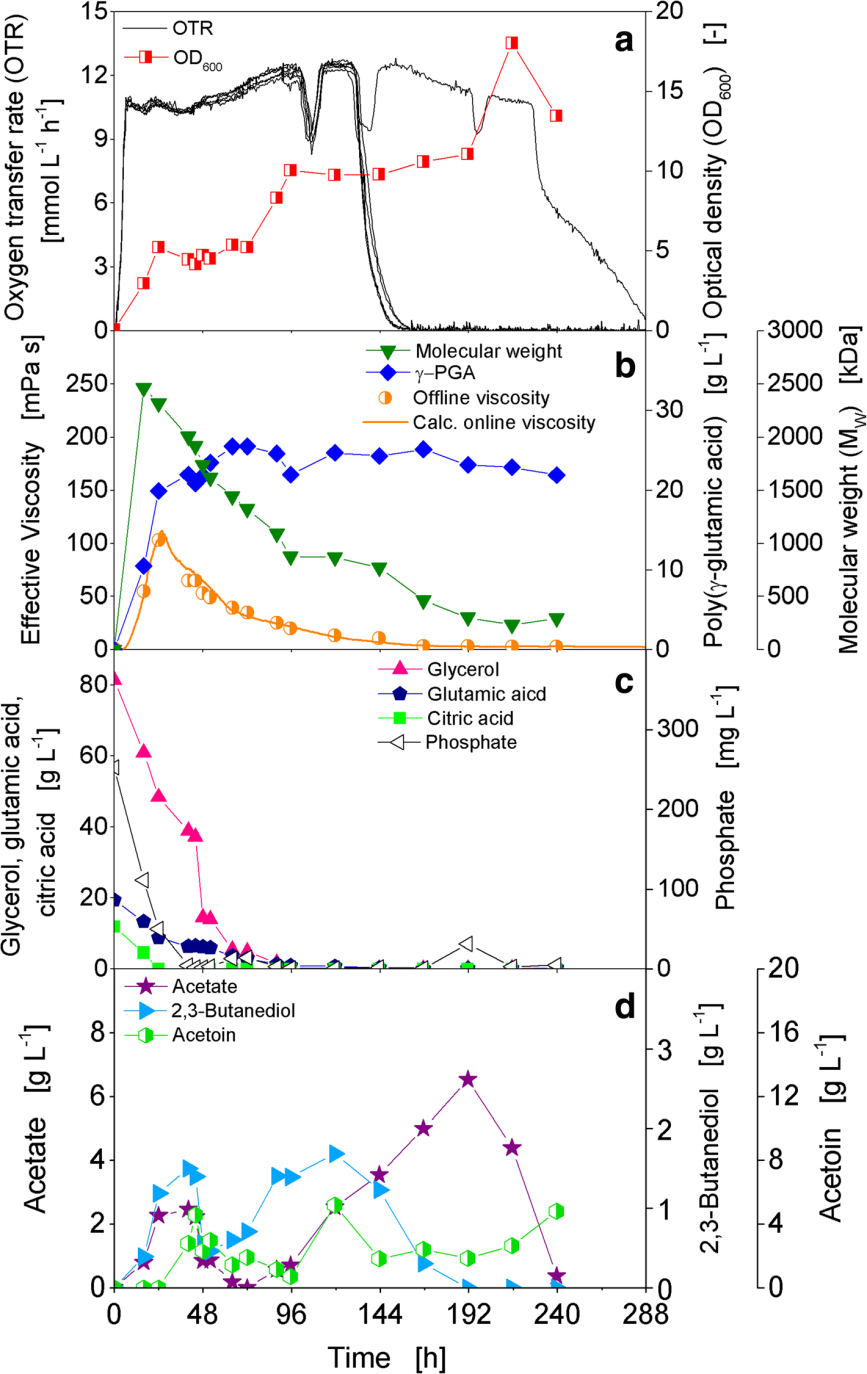
Growth and γ-PGA production of *B. licheniformis* ATCC 9945 in standard mineral medium E. **a** Oxygen transfer rates (OTR) of six parallel flasks and optical density (OD_600_). **b** Derived online viscosity, measured offline viscosity, molecular weight, and concentration of γ-PGA. **c** Carbon source concentrations of glycerol, glutamic acid, and citric acid, and phosphate concentration. **d** Overflow metabolite concentrations of acetate, acetoin, and 2,3-butanediol. Initial values: pH = 7.4, 80 g L^−1^ glycerol, 20 g L^−1^ glutamic acid, 12 g L^−1^ citric acid. Cultivation conditions: *T* = 37 °C, 500 mL shake flasks, filling volume 100 mL, shaking frequency 250 rpm, shaking diameter 50 mm

The strain *B. licheniformis* ATCC 9945 is one of the two strains used for the experiments presented in this study to investigate γ-PGA production with online power input measurement. The strain ATCC 9945 is a known γ-PGA producer: multiple publications are available about this strain [28, 21, 29–33]. Figure 3 displays the results of a shake flask cultivation of *B. licheniformis* ATCC 9945 in standard mineral medium E [21] with shaking conditions similar or identical to those often found published for production of γ-PGA [31, 32, 34]. Figure 3a shows the OTRs of six parallel RAMOS shake flasks. The OTRs increased exponentially up to 11 mmol L^−1^ h^−1^ and reached a horizontal plateau after 6 h which indicates an oxygen limitation [24]. After about 130 h, the OTRs of five of the six parallel flasks decreased, reaching 0 mmol L^−1^ h^−1^ after 155 h. The OTR of one of the flasks continued on the oxygen limited plateau and finally decreased after 225 h to reach 0 mmol L^−1^ h^−1^ after 290 h. For all six shake flasks, a smaller drop in the OTR to about 9 mmol L^−1^ h^−1^ is visible after 100 h. For the single flask which still showed respiration activity after 130 h, such drops in the OTR also occurred after 130 h and 193 h. The optical density increased constantly during cultivation to a maximum of 18 after 215 h, thereafter it declined to a final value of 13.5 (Fig. 3a).

A deviation of the OTR curves of parallel cultivated shake flasks was observed also in other experiments conducted under standard conditions. Only the time point of the diverging varied, occurring at the earliest after about 60 h of cultivation. Additional file 2: Figure S2 (supplementary material) depicts the OTR curves of all cultivations performed during this study under standard conditions in one diagram. The phenomenon of the deviating OTRs could easily be observed with the RAMOS device but would have been more difficult or even impossible to identify if only offline sampling would have been performed. The pH value proved to be the only reliable offline indicator to determine whether a culture had terminated the respiration activity during cultivation or only at the end. It was observed that cultures in RAMOS shake flasks which showed an early decrease in OTR had a final pH value around 5.5. Shake flasks with OTR decreases after 216 h exhibited pH values generally higher than pH 9. After this correlation had been identified, it was also possible to decide whether the cultures of the shake flasks used for offline analysis belonged to the first (low final pH = early termination of respiration activity) or second group (high final pH = late termination). In the experiment depicted in Fig. 3, the final pH values of the five flasks with an earlier decrease of the OTR were between 5.3 to 5.9, while the final pH value of the remaining flask was above 9.9. A deviation of OTR curves was also observed in other experiments of strain ATCC 9945 presented in this work. Therefore, possible reasons for this phenomenon are discussed at large in a separate subchapter after the description and discussion of the other experiments.

The online viscosity calculated from the power input signal showed a strong increase during the first 24 h of cultivation (Fig. 3b). The sharp maximum of 112 mPa s was obtained after 26 h. Afterwards, the online viscosity significantly decreased again and reached a level below 4 mPa s after 168 h, on which it remained until the end of cultivation. The calculated online viscosity is nicely validated by the offline measured effective viscosity values (Fig. 3b). In this study, the measurement of power input and viscosity, respectively, during the cultivations was applied as a tool for continuous online measurement of γ-PGA production. However, since the viscosity of a γ-PGA containing solution is influenced by the molecular weight as well as the concentration, gel permeation chromatography was also applied to enable the measurement of both parameters separately. With this technique, it was possible to quantify the changes in molecular weight and concentration of γ-PGA during cultivations, and to compare these with the trend of the effective viscosity. The maximum of the mass average molecular weight (M_w_) of 2460 kDa was determined at 16 h. Thereafter, it decreased significantly and reached a final value of 300 kDa after 240 h. After about 26 h, when the maximum viscosity was determined, the molecular weight had slightly decreased to 2310 kDa. Interestingly, while the molecular weight decreased, the γ-PGA concentration still increased. At 16 h, the concentration was only 10.5 g L^−1^, and after 24 h it had reached 19.9 g L^−1^. The maximum concentration of 25.5 g L^−1^ was measured between 64 and 72 h. The final measured concentration after 240 h was 21.9 g L^−1^.

The viscosity of a γ-PGA solution depends on its concentration and molecular weight. It could indirectly also be influenced by the pH. The pH has an impact on the conformation of γ-PGA, e.g. α-helix, β-sheet or random coil [35], which in turn influences the viscosity. The viscosity changes significantly at extreme pH values, e.g. below pH 5 or above pH 8-9, when there is a change in conformation from random coil to α-helix for instance [36]. In the experiments of this study, the pH never fell below pH 5.3 and didn’t rise above pH 8 until at least 120 h of cultivation. According to Ho et al. [36], the viscosity increases with increasing pH. Therefore, if the pH had an influence on the viscosity of γ-PGA as significant as the influence of concentration and molecular weight, an increase in viscosity should be observable towards the end of the cultivation, when the pH increased. But such an increase could not be detected; quite the contrary, the viscosity decreased towards the end of the cultivations, due to a decrease in molecular weight or concentration, respectively.

Figure 3c displays the consumption of the three carbon sources glycerol, glutamic acid, and citric acid, as well as the phosphate concentration. All three carbon sources are consumed in parallel. Citric acid is depleted first after 24 h, followed by glutamic acid and glycerol, whose concentrations had decreased below 1 g L^−1^ at 96 h. The concentrations of ammonium (data not shown) and phosphate were also measured to determine whether the nitrogen or phosphate source might become limiting during cultivation. Starting at a concentration of approximately 2700 mg L^−1^, ammonium decreased strongly during the first 24 h but didn’t become limiting at any time during the cultivation. The initial phosphate concentration of 250 mg L^−1^ was depleted after 40 h and remained low until the end of the fermentation. During cultivation, the overflow metabolites acetate, acetoin, and 2,3-butanediol were formed and also consumed again after the depletion of the initial carbon sources (Fig. 3d). Acetate showed the highest concentration with 6.5 g L^−1^ at 192 h. The highest concentrations of acetoin, 5.2 g L^−1^, and 2,3-butanediol, 1.7 g L^−1^, were obtained at 120 h. Acetate, acetoin, and 2,3-butanediol are typical overflow metabolites of *Bacillus* species [37, 38] and have been detected as typical by-products of γ-PGA production before [31, 39]. The oxygen availability during cultivation influences the ratio of the three overflow metabolites. In case of unlimited oxygen availability, the share of acetate will be the highest since it is the most oxidized metabolite of the three. With decreasing oxygen availability, first the share of acetoin, and second, of 2,3-butanediol will increase. 2,3-Butanediol is the most reduced one of the overflow metabolites [40]. In case of oxygen limited or anaerobic conditions the production of acetoin and especially 2,3-butanediol can be used for the regeneration of NADH^+^ to NAD^+^. Moreover, the interconversion of acetoin and 2,3-butanediol serves as an oxidation-reduction buffer system for NAD^+^/NADH^+^ [37, 40].

This experiment shows that the online viscosity signal calculated from the online measured power input correlates very well with the offline determined effective viscosity values. It is also obvious that the viscosity, and, therefore, also the power input, depends on both, molecular weight and concentration, of the produced γ-PGA. However, changes in the molecular weight seem to have a greater influence than changes in the concentration. For the biopolymer alginate it was shown before that a change in the molecular weight has a significant higher impact on the viscosity than an increase or decrease in concentration [18, 41]. The decrease in molecular weight of γ-PGA is very likely due to the secretion of γ-PGA specific depolymerases. From γ-PGA producing *Bacillus subtilis* strains, two degrading enzymes were reported, one acting in an endopeptidase-like fashion, cleaving the polymer into smaller oligomers [42]. These oligomers can be further cleaved by an exopeptidase into single glutamate monomers [43]. Especially the activity of an endo-depolymerase should have a significant impact on the molecular weight and, thus, on viscosity and power input. The activity of an exo-depolymerase is probably the limiting step in the degradation of the afore-produced γ-PGA to consumable glutamate monomers. After the depletion of the initial amount of glutamic acid, glutamic acid is not detected in the fermentation broth anymore. Moreover, the γ-PGA concentration remained almost constant after reaching the maximum. Thus, the enzymatic degradation of γ-PGA to single, consumable glutamate monomers rather does not take place. The enzymatic degradation of the produced γ-PGA starts, shown by the decrease in molecular weight, already after 16 h, when the carbon sources glycerol and glutamic acid are still present in significant concentrations (61 and 13 g L^−1^, respectively). Therefore, the degradation has to be triggered by something else than the general depletion of carbon source. Also an ammonium limitation as the trigger can be excluded, since the concentration never decreased below 500 mg L^−1^. However, both the citric acid and the phosphate concentration were already quite low at this time. Therefore, the influence of a higher citric acid and a higher phosphate concentration on γ-PGA formation was investigated. An increase of the phosphate concentration by a factor of three showed no influence (data not shown). The phosphate limitation, thus, seems to have neither a negative nor a positive influence on γ-PGA production. In the next paragraph, the influence of a higher citric acid concentration regarding its impact not only on viscosity, but also on γ-PGA molecular weight and concentration is discussed.

### Cultivation of *Bacillus licheniformis* ATCC 9945 in mineral medium E with 1.5× citric acid

**Fig. 4:**
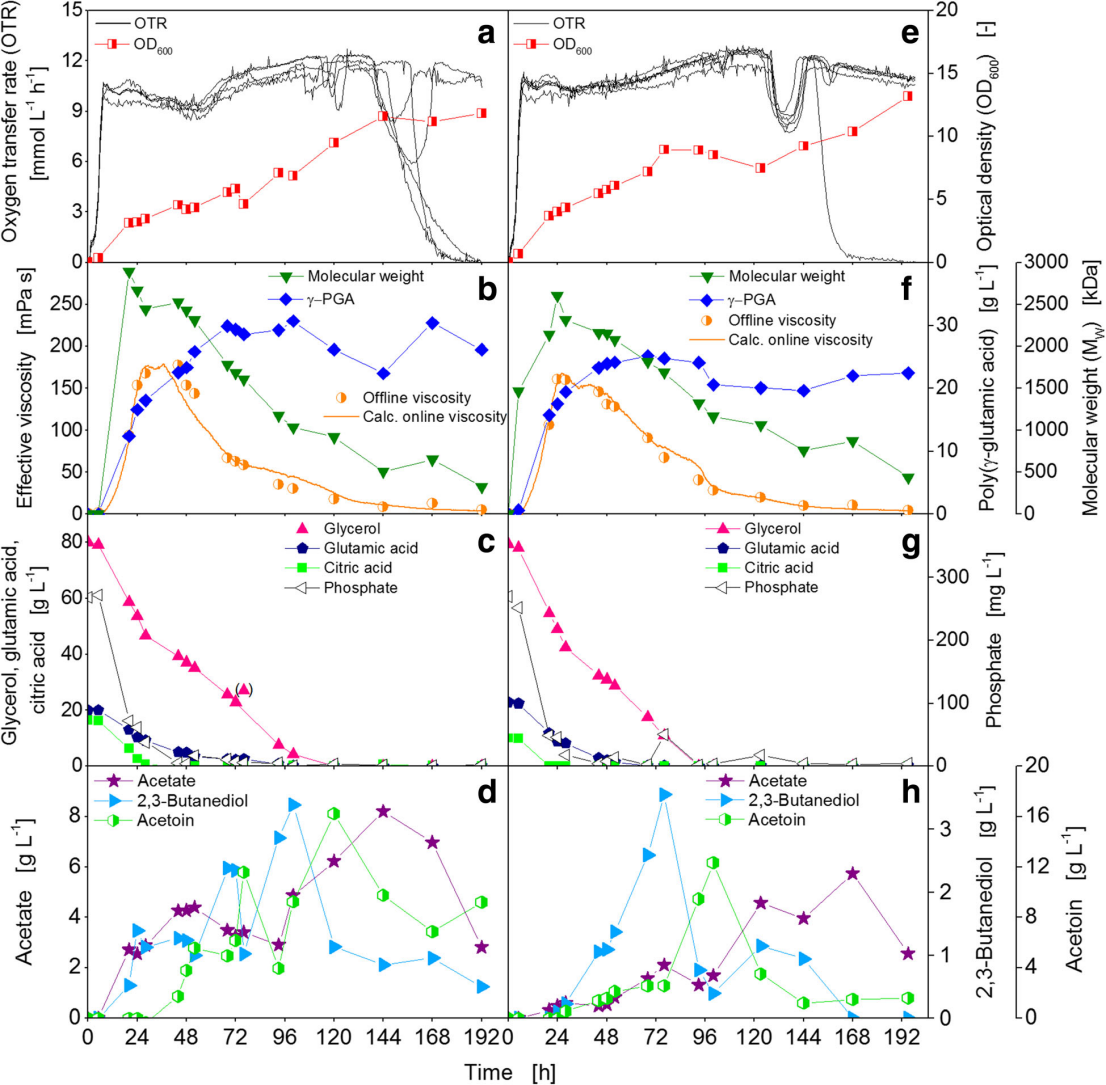
Impact of additional citric acid (**a-d**) or additional trace elements (**e-h**) on γ-PGA production of *B. licheniformis* ATCC 9945. **a** and **e** Oxygen transfer rates (OTR) of five and six parallel flasks and optical density (OD_600_). **b** and **f** Derived online viscosity, measured offline viscosity, molecular weight, and concentration of γ-PGA. **c** and **g** Carbon source concentrations of glycerol, glutamic acid, and citric acid, and phosphate concentration. **d** and **h** Overflow metabolite concentrations of acetate, acetoin, and 2,3-butanediol. Initial values: pH = 7.4, 80 g L^−1^ glycerol, 20 g L^−1^ glutamic acid, 18 **(a-d)** or 12 g L^−1^ **(e-h)** citric acid, trace elements (only **e-h**). Cultivation conditions: *T* = 37 °C, 500 mL shake flasks, filling volume 100 mL, shaking frequency 250 rpm, shaking diameter 50 mm

Figure 4a-d shows a cultivation of *B. licheniformis* ATCC 9945 in mineral medium E with 1.5× citric acid compared to the standard mineral medium E (Fig. 3). The citric acid concentration was increased to investigate its influence on time point and absolute maximal values of γ-PGA molecular weight and concentration. In this experiment, the strain was cultivated for only 192 h compared to 330 h for the standard approach (Fig. 3), since the standard experiment showed that the interesting γ-PGA production and degradation phase takes place in the first approximately 96 to 120 h. Similar to the standard approach, the OTRs of the five parallel cultivated RAMOS shake flasks show almost no lag-phase followed by an exponential increase to 11 mmol L^−1^ h^−1^ (Fig. 4a). From this level, the OTRs continued as a horizontal plateau, indicating the oxygen limitation which was already observed for the standard approach. Between 140 to 155 h, three of the five OTRs started to decrease and reached a value of 0 mmol L^−1^ h^−1^ at 192 h. The other two OTRs showed only a drop to 8.5 and 6 mmol L^−1^ h^−1^, respectively. Apparently, the cultures in these flasks recovered and continued to show respiration activity until the experiment was terminated. Congruent with the standard approach, the shake flasks with an early decrease in OTR had low final pH values of approximately 5.6 while for the flasks with continuing OTRs pH values of 7.0 and 7.7 were measured at the termination of the experiment. The optical density increased steadily from 0.05 up to a value of 11.6 at 144 h where it remained constant.

With additional citric acid, a higher maximum viscosity was measured. The maximum was also shifted slightly to a later time point between 28 and 44 h (Fig. 4b). The highest online viscosity of 179 mPa s was determined after 37 h, which is significantly higher than for the standard approach. Also, a higher M_w_ than for the standard cultivation was determined. The highest M_w_ of 2900 kDa was measured for the sample after 20 h. At the same time, a γ-PGA concentration of 12.4 g L^−1^ was determined. From 20 h onwards, the molecular weight decreased steadily throughout the cultivation to a final value of 330 kDa at 192 h. On the contrary, the γ-PGA concentration further increased to a maximum of 30.7 g L^−1^ at 100 h, which is about 5 g L^−1^ or 20% more than in the standard approach. A positive influence of higher citric acid concentrations in medium E was also recently demonstrated by Mitsunaga et al. [39]. In that work, also a higher γ-PGA concentration was reported for an increase of the citric acid concentration by the factor 1.5. However, no data for M_w_ were shown. Furthermore, citric acid might not only serve as the preferred carbon source for γ-PGA production, but is probably also working as an effective chelator of metal ions. As a chelator, citric acid would have a double function. First, it generally makes important trace metal ions biologically available for *B. licheniformis*, as also suggested by Ko and Gross [44]. Second, the chelation of essential metal ions in the medium by citric acid prevents that these ions are chelated by the produced γ-PGA. γ-PGA effectively binds positive charged metal ions which subsequently are no longer available for *B. licheniformis* [7, 45].

Despite the increase of the initial concentration, citric acid was still the first carbon source which was depleted, however, slightly later than in the standard cultivation. Glutamic acid was depleted after 96 h and glycerol after 120 h. The phosphate consumption was very similar to the standard cultivation (Fig. 4c). The overflow metabolites acetate, acetoin, and 2,3-butanediol were produced with maximum concentrations of 8.2, 16.2, and 3.4 g L^−1^, respectively. Acetate production was in the same order of magnitude as for the standard cultivation. However, acetoin and 2,3-butanediol formation was significantly higher (Fig. 4d).

The increase of citric acid by 50% (*w*/*v*) led to a significant increase in effective viscosity and volumetric power input. This increase was caused by an increased M_w_ as well as a higher maximum concentration of γ-PGA. This demonstrates that a difference in the γ-PGA production due to a different medium composition can reliably be detected with online power input measurement and calculation of the online viscosity, respectively.

### Cultivation of *Bacillus licheniformis* ATCC 9945 in mineral medium E with trace elements

Figure 4e-h shows a cultivation of *B. licheniformis* ATCC 9945 in mineral medium E with additional trace elements. Trace elements were supplemented to investigate whether the medium lacks certain micronutrients which are important for the γ-PGA production or if the lack of certain elements is involved in triggering the enzymatic γ-PGA degradation. In general, the OTRs curves of the six parallel cultivated RAMOS shake flasks displayed the same overall progression as the OTR curves from the experiments discussed above. Yet, the single OTRs ran more in parallel. All flasks show a drop in the OTR between 125 and 145 h. The OTR of only one flask decreased completely after about 150 h while all other flasks showed continuous respiration activity until the experiment was terminated at 192 h (Fig. 4e). The final pH values of the cultures in the RAMOS flasks also correlated with this observation. The maximal OD of 13.2 was measured after 192 h, which is slightly higher than for the other two conditions at the same time point.

The maximum viscosity calculated from the online power input signal was 168 mPa s, compared to 112 mPa s in the standard medium E without trace elements. The maximum was measured after the same time as in the standard cultivation, namely after 26 h (Fig. 4f). The highest M_w_ of 2600 kDa was determined offline at 24 h, and, therefore, slightly later than in the standard cultivation. The γ-PGA concentration was 17.5 g L^−1^ at this time point and increased to a maximum of 25.1 g L^−1^ at 68 h. Similar to the standard cultivation and to the experiment with 1.5× citric acid, the γ-PGA concentration afterwards remained nearly constant. The carbon sources citric acid, glutamic acid, and glycerol were depleted after 20, 68, and 93 h, respectively, which is similar to the standard cultivation regarding citric acid and glycerol. Glutamic acid was depleted approximately 24 h sooner in the medium with trace elements compared to the standard medium. The phosphate consumption was similar to the standard cultivation. The initial phosphate concentration was depleted at 44 h (Fig. 4g). The overflow metabolites acetate, acetoin, and 2,3-butanediol were produced in maximum concentrations of 5.7, 12.3, and 3.6 g L^−1^. Acetate production was similar to the standard approach, but more acetoin and 2,3-butanediol were formed. This is similar to the cultivation with 1.5× citric acid (Fig. 4h).

The simple addition of trace elements such as cobalt, zinc, nickel, and copper resulted in a distinctly higher effective viscosity and volumetric power input due to a higher M_w_. Moreover, the OTRs of the parallel running cultures showed a more uniform behavior than in the experiments without trace elements. This suggests that in standard mineral medium E, as originally published by Leonard et al. [21], some trace elements are missing to support unlimited growth and production. Leonard et al. also investigated the influence of cobalt and zinc on γ-PGA production and cell viability of *B licheniformis* ATCC 9945. They found that varying concentrations of both ions influenced the D- to L-glutamate isomer ratio in the produced γ-PGA but did not increase γ-PGA concentration or cell viability. They didn’t include copper and zinc in the their final medium composition. However, they also stated that although a requirement such as for zinc could not be confirmed in their experiments, they could not rule out that sufficient zinc was already provided by contamination of other medium ingredients. Thus, either other trace elements are limiting in medium E, for instance copper, which was not tested by Leonard et al.; or zinc and cobalt were indeed provided by contamination of other medium ingredients in their experiments [21]. It has to be kept in mind, that five decades ago, medium compounds were probably not produced with such a level of purity as today and, therefore, might have contained more trace elements. More recently, it was demonstrated that zinc boosts the activity of the γ-PGA synthesis complex in vitro [46]. Therefore, a supplementation with this trace element might also increase γ-PGA production in vivo.

### Cultivation of *Bacillus licheniformis* A1 in mineral medium E

**Fig. 5:**
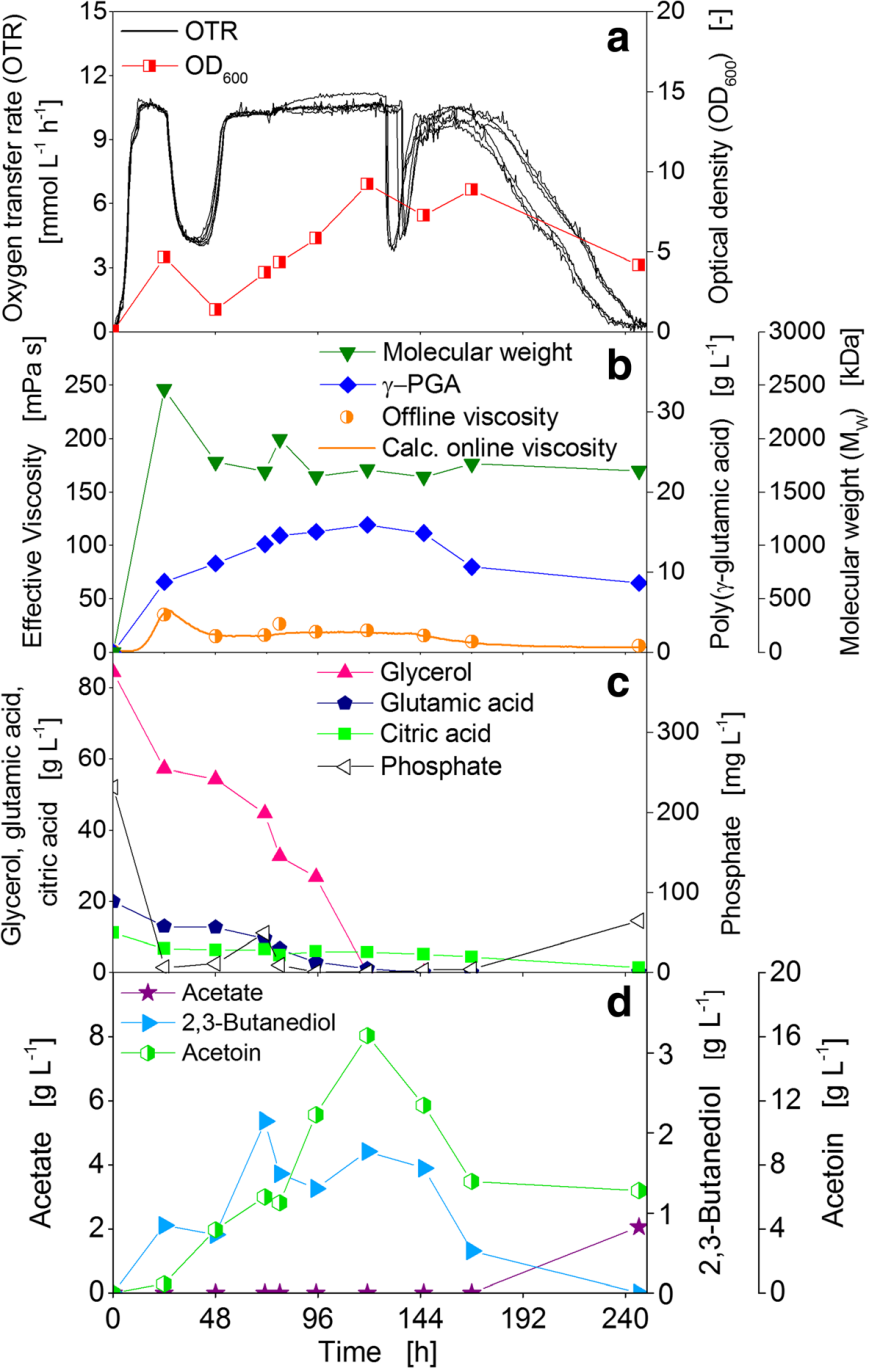
Growth and γ-PGA production of *B. licheniformis* A1 in standard mineral medium E. **a** Oxygen transfer rates (OTR) of six parallel flasks and optical density (OD_600_). **b** Derived online viscosity, measured offline viscosity, molecular weight, and concentration of γ-PGA. **c** Carbon source concentrations of glycerol, glutamic acid, and citric acid, and phosphate concentration. **d** Overflow metabolite concentrations of acetate, acetoin, and 2,3-butanediol. Initial values: pH = 7.4, 80 g L^−1^ glycerol, 20 g L^−1^ glutamic acid, 12 g L^−1^ citric acid, kanamycin. Cultivation conditions: *T* = 37 °C, 500 mL shake flasks, filling volume 100 mL, shaking frequency 250 rpm, shaking diameter 50 mm

A second γ-PGA producing strain, *B. licheniformis* A1, was cultivated in standard mineral medium E (with kanamycin) to investigate how different strains vary in their growth and production behavior and if the differences can be detected with the online signals of OTR and volumetric power input. Figure 5 shows the results of the cultivation of strain A1. Identical to strain ATCC 9945, the strain A1 shows exponential growth after a very short lag-phase. Upon reaching an OTR level of 11 mmol L^−1^ h^−1^, the cultures entered into an oxygen limitation indicated by the constant OTR level. Between 25 to 55 h the OTRs of all six parallel RAMOS flasks showed a drop to about 4 mmol L^−1^ h^−1^. The OTR plateau then remained constant until about 130 h when the OTRs show another drop, also down to 4 mmol L^−1^ h^−1^. After 176 h, the OTRs started to decline slowly and reached 0 mmol L^−1^ h^−1^ after 240 h. The six parallel cultures of strain A1 show a much more reproducible respiration activity than strain ATCC 9945 in the previous experiments. This reproducibility of the OTR curves is also reflected by the final pH values in the RAMOS flasks which were all above 9.2. The progression of the OD correlates with the OTR. In general, the OD is increasing during the cultivation, but parallel to the drops in OTR also drops in optical density were observed. The highest OD of 9.2 was determined after 120 h. In this experiment, strain A1 showed a significantly lower viscosity than strain ATCC 9945 under the same conditions. A maximum effective viscosity of 39 mPa s and a maximum M_w_ of 2460 kDa were determined at the same time point. The γ-PGA concentration was 8.8 g L^−1^ at 24 h, increased to its maximum of 15.9 g L^−1^ at 120 h, and decreased to 8.7 g L^−1^ until the end of the cultivation.

The carbon source consumption was different compared to strain ATCC 9945. Glycerol and glutamic acid were both depleted after 120 h. Citric acid, however, was only slowly consumed. At the end of the experiment, 1.4 g L^−1^ were still measured. This is in clear contrast to strain ATCC 9945, where in all experiments citric acid was the first carbon source to be depleted and which had a measurable impact on γ-PGA production. Overflow formation of acetate and acetoin also differed between the two strains. Acetate was only formed towards the end of the cultivation with a concentration of 2.1 g L^−1^ measured at 246 h. The highest acetoin concentration of 16.1 g L^−1^ was measured at 120 h which correlates with the time point of glycerol and glutamic acid depletion. Part of the acetoin was consumed again and a final concentration of 6.4 g L^−1^ was obtained. However, 2,3-butanediol formation was in the same order of magnitude for both strains. A maximum concentration of 2.2 g L^−1^ was determined after 72 h. The formed 2,3-butanediol was completely consumed until the end of the cultivation. This experiment clearly demonstrated the differences in growth and product formation of two γ-PGA producing *B. licheniformis* strains.

### Deviating OTR curves of *B. licheniformis* ATCC 9945

In parallel cultures of *B. licheniformis* ATCC 9945 some cultures fail to cope with a low pH value and others are able to recover and cause an increase of pH value, leading to deviating OTR curves as shown in Figs. 3, 4, and S2. It is suggested that the divergence of the supposedly identical cultures is due to the development of various cell types or subpopulations in the cultures. Minor differences during the cultivation might subsequently result in the major divergence of respiration activities. An event, which would result in different subpopulations, is the induction of spore formation. For different sporulating *Bacillus* species the application of flow cytometry has revealed heterogeneous populations in batch and chemostat cultures [47–49]. The applied *B. licheniformis* strain ATCC 9945 is a wildtype strain and harbors active sporulation genes. For the wildtype *B. subtilis* strain Marburg, it was observed that in exponentially growing cultures, the bacterial population consisted of three types: growing cells, non-growing cells committed to sporulate, and spores [50]. In this work, spore formation was also observed during cultivation by taking microscopic pictures of the cultures. Often, spores were observed for the first time after approximately 40 h, but sometimes already after 24 h. The number of spores increased steadily during cultivation, from very few spores inside the cells, to more frequent spores inside the cells and also free spores. Hence, the sporulation process should seriously be considered as a reason for the divergence in respiration activities. For *B. licheniformis*, it was observed that different cell morphologies existed in the same culture on agar plates. The cells differed in their size and motility: short, motile cells with flagella and chains of cells which were non-motile [51]. For the cultures in this work, also different cell types were observed by microscopy. At the beginning of cultivations, predominantly short cells and a few longer cells were present. Subsequently, less short and more long cells appeared. Short cells often were present as single cells, while the longer cells existed mainly as doubles or chains of cells. Bisset and Street [51] also noticed that spores were produced more frequently in the short cells and only rarely in the filamentous-like cells. In this work, both aspects – sporulation and different cell types – were observed for the cultures of *B. licheniformis* ATCC 9945. To answer the question whether one or both of these aspects are responsible for the divergence of the cultures, it is useful to take a look at a non-sporulating *B. licheniformis* strain. In strain A1, one important gene for the sporulation process, *spo0A*, was deleted, and sporulation thereby inactivated. Cultivations of A1 under standard conditions showed much more reproducible curves of respiration activity (Fig. 5). The final pH values of the cultures were usually above 9; there were only very few exemptions of cultures with low final pH values. The cultures of A1 also showed different cell types during cultivation. All these observations suggest that spore formation, and the gene regulation associated with the whole process of spore formation, might be responsible for or at least participates in the divergence of the cultures, resulting ultimately in the early termination of respiration activity of some of them.

### Comparison of online viscosity signals of *B. licheniformis* ATCC 9945 and A1 in different variations of mineral medium E

**Fig. 6:**
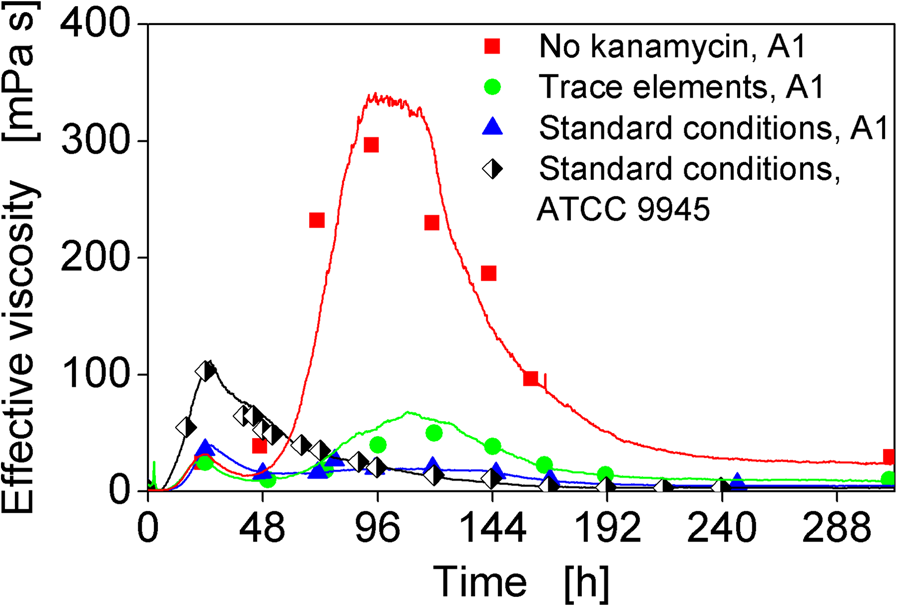
Online and offline viscosity measurements of *B. licheniformis* A1 and ATCC 9945 cultivations in different mineral medium E compositions. Initial values: pH = 7.4, 80 g L^−1^ glycerol, 20 g L^−1^ glutamic acid, 12 g L^−1^ citric acid, trace elements and/or kanamycin. Cultivation conditions: *T* = 37 °C, 500 mL shake flasks, filling volume 100 mL, shaking frequency 250 rpm, shaking diameter 50 mm

The strain A1 of *B. licheniformis* was not only characterized under standard conditions in mineral medium E, but was also investigated in different medium compositions. The supplementation with trace elements had shown a positive effect on γ-PGA production of strain ATCC 9945. Therefore, the supplementation of trace elements was investigated to determine whether the lower γ-PGA production of strain A1 is due to a limitation of trace elements. Strain A1 was usually cultivated with the antibiotic kanamycin, since it carries a plasmid with the respective resistance gene. However, experiments with this strain conducted during this work suggested that the antibiotic might have a negative influence on the γ-PGA production. Therefore, in addition to a supplementation with trace elements also a cultivation without kanamycin was conducted to determine whether kanamycin has an influence on the γ-PGA production of strain A1. Figure 6 shows online and offline viscosity measurements for different cultivations of *B. licheniformis* A1: in standard medium E with kanamycin, in medium E with trace elements and kanamycin, and in standard medium E without kanamycin. For comparison, the results of strain ATCC 9945 in standard medium E are also depicted.

For all cultivations, a (local) maximum at about 24 h was observed. Interestingly, for strain A1 in medium E with trace elements and in medium E without kanamycin, the actual maximum viscosity was measured much later in the cultivation, namely after 108 and 95 h, respectively. This progression is also reflected in the data of the offline measured viscosity. For the cultivation of strain A1 in standard medium E without kanamycin, the highest online as well as offline viscosities of all experiments were determined, which were 341 and 297 mPa s at 95 and 93 h. Obviously, in case of strain A1 the antibiotic kanamycin has a strong impact on γ-PGA production despite the strain’s genetic resistance.

### Measured and calculated viscosity

**Fig. 7:**
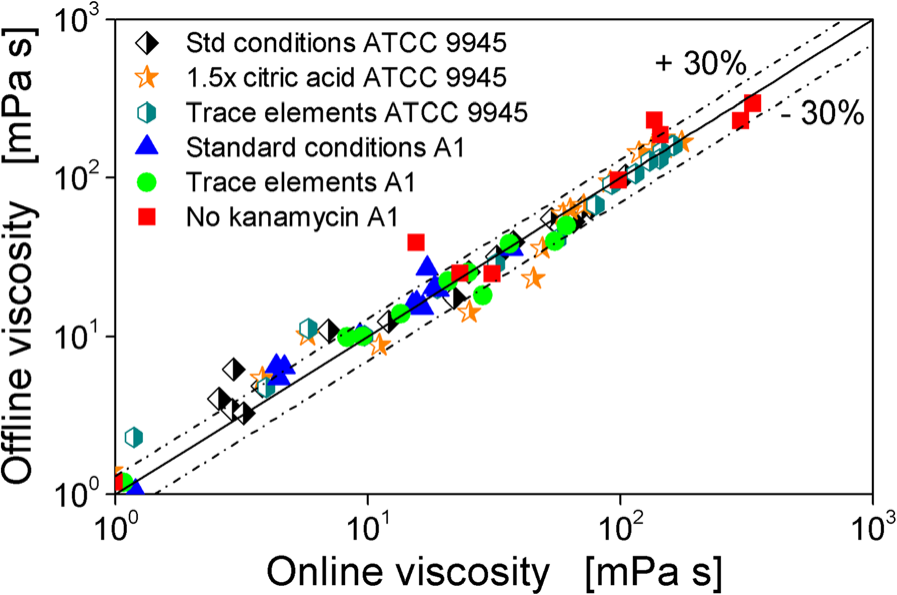
Measured (offline) and calculated (online) viscosity of all cultivations presented in this work. The offline effective viscosity was obtained via rheometer measurements of sampled cultivation broth. The online viscosity was calculated from the volumetric power input measured online via the power input shaker. Initial values: pH = 7.4, 80 g L^−1^ glycerol, 20 g L^−1^ glutamic acid, 12 g L^−1^ citric acid, trace elements and/or kanamycin. Cultivation conditions: *T* = 37 °C, 500 mL shake flasks, filling volume 100 mL, shaking frequency 250 rpm, shaking diameter 50 mm

In Fig. 7, measured values of viscosity (offline viscosity) are plotted against the values calculated by Eq. (2), (4), and (5) from the online power input signal. It is obvious, that almost all measured results lie within a range of ±30% tolerance. This demonstrates that the online measured volumetric power input can reliably be applied to derive an online viscosity signal to follow γ-PGA formation and degradation in shake flask cultures of *B. licheniformis*.

## Conclusions

This study demonstrates the application of volumetric power input measurement for shake flask cultivations to follow γ-PGA formation and degradation. For these investigations, a *B. licheniformis* γ-PGA production strain (ATCC 9945) and a protease producing *B. licheniformis* strain (A1) with an undesired capability of γ-PGA formation were analyzed and compared regarding their growth and production behavior. It was shown that online measurement techniques for the determination of volumetric power input and OTR are valuable tools to characterize and optimize γ-PGA production by *B. licheniformis*. The volumetric power input was used to derive an online viscosity signal which correlated very well with offline measured viscosity values. Online power input and online viscosity, respectively, can effectively be used to follow γ-PGA production and degradation. OTR measurements are useful to evaluate the general behavior and reproducibility of respiration activity. Power input and viscosity depended on molecular weight and concentration of the produced γ-PGA, but changes of the molecular weight had a higher influence than changes of the concentration.

An increase of the citric acid concentration in the medium led to a significant increase of viscosity. Moreover, an increase was observed for the maximum M_w_ as well as for the maximum concentration of γ-PGA. This demonstrated that a difference in γ-PGA production can be detected with online viscosity measurements. The addition of trace elements resulted also in a distinctly higher viscosity, this time caused by an increase in M_w_ only. The OTRs showed a more reproducible respiration activity for parallel cultures under these conditions. This suggests that in standard mineral medium E, as originally published by Leonard et al. [21], certain trace elements are missing to support unlimited growth and production.

The investigation of two γ-PGA producing strains illustrated that differences between strains can be detected with the presented online techniques. For strain A1 the maximum viscosity were observed much later than for the strain ATCC 9945 under certain conditions. For the cultivation of strain A1 in standard medium E without kanamycin, the highest effective viscosity of all experiments was determined, which was 341 mPa s.

## Additional files

Additional file 1: Figure S1. Online measured volumetric power input and calculated online viscosity of a cultivation of *B. licheniformis* ATCC 9945 in standard mineral medium E. The online viscosity signal was calculated from the measured power input with Eq. (2), (4), and (5). Cultivation conditions: Medium E, *T* = 37 °C, 500 mL shake flasks, filling volume 100 mL, shaking frequency 250 rpm, shaking diameter 50 mm. (TIFF 32 kb)

Additional file 2: Figure S2. OTR curves of *B. licheniformis* ATCC 9945 cultivations in standard mineral medium E. OTR curves of 25 RAMOS shake flasks from eight individual experiments. Each colour represents the curves of one experiment. Deviating OTR curves were observed in all of these experiments. Initial values: pH = 7.4, 80 g L^−1^ glycerol, 20 g L^−1^ glutamic acid, 12 g L^−1^ citric acid, 7 g L^−1^ NH_4_Cl, 0.5 g L^−1^ K_2_HPO_4_. Cultivation conditions: *T* = 37 °C, 500 mL shake flasks, filling volume 100 mL, shaking frequency 250 rpm, shaking diameter 50 mm, inoculation from glycerol stock (0.4% *v*/v). (TIFF 96 kb)

## Abbreviations

B. licheniformis: Bacillus licheniformis
M_w_: mass average molecular weight [kDa]
OD: optical density at 600 nm [−]
OTR: oxygen transfer rate [mmol L^−1^ h^−1^]
RAMOS: Respiration Activity Monitoring System
γ-PGA: poly(γ-glutamic acid)
